# Recombinant CALR as a novel immune adjuvant in tumor immunotherapy

**DOI:** 10.1016/j.omton.2026.201188

**Published:** 2026-04-07

**Authors:** Katelyn Ginley, Shipra Solanki, Rachael A. Connington, Kishore B. Challagundla, Ranjan Solanki

**Affiliations:** 1 DO Program in Interdisciplinary Studies at Touro College of Osteopathic Medicine, Middletown, NY, USA; 2 Clinical Researcher, Goshen, NY, USA; 3 Department of Basic Biomedical Sciences, Touro College of Osteopathic Medicine, Middletown, NY, USA

## Main text

Cold tumors, characterized by limited immune cell infiltration and the absence of a localized inflammatory response, remain largely unresponsive to immune checkpoint inhibitors (ICIs), which are successful in treating other malignancies.^1^ The studies carried out by Manole S. and Forbes N.S. mark an important progress in leveraging the immune system for cancer therapy, particularly in addressing the continuing challenge of immunologically cold tumors.^2^ By investigating recombinant calreticulin (CALR) as a damage-associated molecular pattern (DAMP) to reprogram the tumor microenvironment (TME), this study offers novel insights and suggests promising directions for future immunotherapies.

Immunotherapy has transformed cancer treatment for many patients; however, its therapeutic effect is often restricted to those with hot tumors, which exhibit substantial infiltration of cytotoxic T cells and an immunostimulatory TME. In contrast, cold tumors, which are poorly infiltrated by immune effector cells and frequently dominated by immunosuppressive myeloid populations, remain largely resistant to these therapies.^3^ Tumor-associated macrophages (TAMs), often skewed toward an M2, pro-tumorigenic phenotype in cold tumors, constitute a major obstacle to effective immunotherapy. In contrast, polarization of TAMs toward the M1 phenotype, characterized by pro-inflammatory and antitumorigenic properties, has emerged as a promising strategy to overcome immune resistance.^4^

CALR is a well-established DAMP that acts as an “eat me” signal when exposed on the surface of dying or stressed cells, promoting their uptake by antigen-presenting cells and subsequent activation of adaptive immunity. The authors propose that recombinant CALR, delivered directly to tumors, can drive TAMs toward the M1 phenotype and simulate immunogenic cell death (ICD), thereby creating a pro-inflammatory TME conducive to robust antitumor immune responses.

The strength of this study is its multi-layered experimental approach, which combines *in vitro*, *ex vivo*, and *in vivo* models to methodically evaluate the immunostimulatory properties of recombinant CALR. The use of engineered *Salmonella* to express CALR is particularly innovative, providing a scalable, potentially tumor-targeted platform for DAMP delivery.

*In vitro*, the authors show that CALR treatment robustly polarizes RAW264.7 macrophages toward the M1 phenotype, as indicated by significant increases in inducible nitric oxide synthase (iNOS) expression and upregulation of B7 family co-stimulatory molecules such as CD80 and CD86. These molecules are essential for antigen presentation and effective T cell priming. Importantly, CALR’s effects were compared to other known adjuvants, including heat shock proteins (Hsp70 and GP96), HMGB1, and the clinically used monophosphoryl lipid A (MPL). CALR was found to outperform these alternatives in inducing macrophage polarization, highlighting its unique potency as an immune adjuvant.^2^

The study further demonstrates that conditioned media from CALR-stimulated macrophages are capable of activating dendritic cells, as assessed by increased expression of B7 co-stimulatory markers. This finding spotlights the importance of macrophage-dendritic cell crosstalk in molding the immune response within the TME. Of note, CALR also directly activated dendritic cells, implying that its immunostimulatory effects are not limited to macrophages.

*In vivo*, repeated intratumoral injection of CALR-expressing bacterial lysate into CT26 colon carcinoma tumors in mice resulted in significant tumor growth inhibition, increased infiltration of leukocytes (including M1 macrophages), and a higher number of activated helper T cells relative to controls. The reduction in tumor growth is ascribed to enhanced macrophage polarization and helper T cell activation, which together drive a coordinated antitumor immune response.

One of the major strengths of this study is its head-to-head comparison of CALR with both established DAMPs and clinically approved adjuvants. The superior activity of CALR, especially compared to MPL, further supports its clinical potential. Furthermore, the demonstration that CALR-activated macrophages can stimulate dendritic cell maturation and B7 co-stimulatory molecule expression links innate and adaptive immunity, an essential requirement for durable antitumor responses.

The use of the CT26 syngeneic mouse model, which is known for high immune cell infiltration and responsiveness to immunomodulation, is a reasonable choice for proof-of-concept studies.^5^ However, this choice does limit broad applicability. It remains to be seen whether recombinant CALR will have similar efficacy in other tumor types, especially those with a more pronounced immunosuppressive microenvironment, such as pancreatic or certain brain tumors.

The study’s safety findings are reassuring that no adverse effects were observed in mice. However, translation to the clinic will require rigorous safety evaluation, as DAMPs could theoretically trigger off-target inflammation or systemic autoimmunity. Additionally, while both bacterial lysate and CALR contributed to immune activation, the precise contribution of non-CALR bacterial components (e.g., PAMPs) deserves further scrutiny, particularly as bacterially derived therapies move closer to clinical application.

The authors also highlight that their approach does not directly compare CALR with ICIs *in vivo*, nor does it fully dissect the formation of long-term memory or antigen spread. However, they have observed changes in the T cells infiltrating the tumor and possibly serving the purpose. These represent valuable future directions, as the ultimate goal of cancer immunotherapy is not only tumor regression but also the prevention of relapse via durable immune memory.

Mechanistically, the findings align with the emerging understanding of the TME as a dynamic immunological ecosystem. CALR-induced M1 polarization leads to increased nitric oxide and cytokine production, which in turn promote vascular permeability and recruitment of additional immune cells.^6^ The upregulation of CD80 and CD86 on both macrophages and dendritic cells is critical for the co-stimulation and activation of T cells, which are the main effectors of adaptive antitumor immunity.

The observed increase in activated helper T cells (CD69^+^ and CD4^+^) following CALR administration is notably remarkable. Activated CD4^+^ T cells provide essential help to cytotoxic T lymphocytes and also support the formation of tertiary lymphoid structures within tumors, both of which are associated with improved immunotherapy outcomes.^7^

The authors prudently propose several future research directions, including evaluating CALR in additional tumor models, exploring combination regimens with checkpoint inhibitors, and using engineered bacterial vectors to co-deliver CALR along with tumor antigens. Given that CALR enhances antigen presentation and T cell activation, there is a strong rationale for combining it with checkpoint blockade to achieve synergistic effects.^7^ Furthermore, the integration of CALR with modalities like radiotherapy, which itself can induce ICD, may further heighten its immunostimulatory capacity.

Another important avenue is rigorous assessment of the duration and stability of CALR-induced macrophage polarization, as well as the capacity to generate durable memory T cell responses. These endpoints are critical for translating short-term tumor control into long-term clinical benefit.

In conclusion, the investigators provide robust preclinical evidence that recombinant CALR is a powerful immune adjuvant, capable of reprogramming the TME from immune excluded to immune active. By promoting M1 macrophage polarization, enhancing antigen presentation, and stimulating adaptive immunity, CALR stands out as a promising candidate for improving cancer immunotherapy, particularly in patients with cold tumors. Although further studies are needed to confirm its safety, durability, and synergy with other immunotherapies across diverse tumor settings, this research represents a significant advance in efforts to broaden the reach of immunotherapy to more patients.

**Figure 1:**
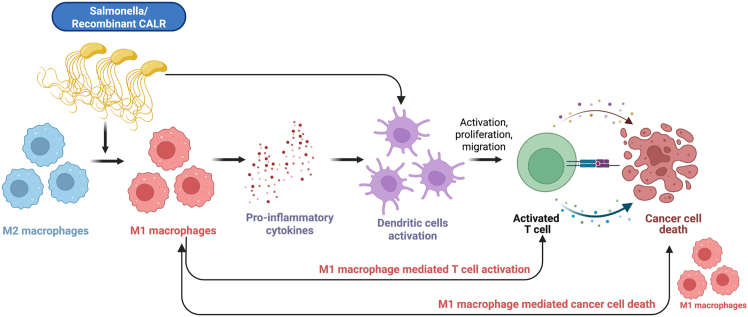
A model illustrating the mechanism by which CALR functions as an immune adjuvant in tumor immunotherapy

A model illustrating the mechanism by which CALR functions as an immune adjuvant in tumor immunotherapy is depicted in Figure 1.

## Declaration of interests

The authors declare no competing interests.
